# What good are psychedelic humanities?

**DOI:** 10.3389/fpsyg.2023.1082933

**Published:** 2023-01-19

**Authors:** Nicolas Langlitz

**Affiliations:** ^1^Department of Anthropology, The New School, New York City, NY, United States; ^2^Institute for Music and Media, Robert Schumann University of Music and Media, Düsseldorf, Germany

**Keywords:** psychedelics, humanities, philosophy, bioethics, humanities laboratory, anthropology, history

## Abstract

The revival of psychedelic research has been dominated by the biomedical sciences. Yet it raises questions that cannot be answered by laboratory experiments and clinical trials alone. Among these are questions pertaining to the conceptual and practical frameworks that render experimental and clinical findings meaningful. Psychedelic humanities clarify the historical presuppositions, philosophical blind spots, and political stakes of different approaches to psychedelics. In this emergent field, many scholars evaluate such alternatives epistemologically, ethically, or politically. However, they could just as well refrain from offering normative orientation and instead increase the complexity of the observed phenomena by opening other possible perspectives, leaving it to their readers to reduce the resulting complexity in novel ways. This enables clinical psychiatrists, laboratory scientists, and other practitioners to use (or abuse) psychedelic humanities scholarship for their own purposes. The article concludes with a note on the institutionalization of such collaboration at The New School’s Psychedelic Humanities Lab.

## Introduction

Since the 1990s, there has been a revival of psychedelic research, which – until the 2010s – was largely confined to the biomedical sciences (Langlitz, 2012a). However, psychedelic experiences raise many questions that cannot be answered by laboratory experiments or clinical trials alone. Laboratory experiments or clinical trials also cannot answer questions about their own place in the larger knowledge culture emerging around psychedelic drugs. Nor can Western societies fall back on old customs to respond to the substances’ expanding medical and nonmedical applications. Instead, they need to think for themselves what psychedelic experiences mean and what their value to late modern life is. Questions of meaning and value are the subject matter of the humanities. Hence, it comes as no surprise that, in the past 15 years, the transition from preclinical research to clinical research has been accompanied by a rise of humanities scholarship on psychedelics. What good are these budding psychedelic humanities?

Note that “the humanities” are not one thing. The empirical research that historians conduct in archives has little in common with the conceptual work that philosophers do in their armchairs. A philologist and a queer theorist will approach one and the same text in radically different manners. The psychedelic humanities also comprise many different styles of thinking and doing. They are asking different questions, which require different methods. But they also articulate different answers to the same question. In the biomedical sciences, controversy indicates that a fact has not yet been fully established. Once it has, controversy will come to an end. Some humanities scholarship also establishes standards that allow no falling back. For example, the discovery of a new set of historical documents or a logical flaw in a philosophical argument can create such milestones. But, for the most part, the humanities do not aim at the kind of durable consensus that we associate with scientific facts. History needs to be rewritten as it proceeds, classical literature allows for multiple interpretations and reinterpretations, and philosophers will continue to disagree over moral and epistemological principles. Such perspectival pluralism is what the psychedelic humanities have to offer to liberal democratic societies that are reevaluating psychedelics without the guardrails of tradition (Langlitz, 2019).

## Enlightened Perspectivism

This perspective on the psychedelic humanities also only marks one of many possible perspectives and makes certain determinations rather than others. It considers the psychedelic humanities as part of what social theorist Niklas Luhmann called the Sociological Enlightenment. The Enlightenment of the 17th and 18th centuries promoted rationality, the empirical observation of the world, as well as moral and political progress. By contrast, the Sociological Enlightenment of the 19th and 20th centuries promoted the observation of other observers, which reveals their standards of rationality, empiricism, morality, and politics to be contingent on the observers’ standpoints – and these standpoints to be contingent on their historical situations and cultural contexts (Luhmann, 1998; Rabinow, 2008). The humanities do not necessarily determine what is or should be the case but can also examine how others make such determinations. In eyes of Luhmann, the key question was which conceptual distinctions were used in the process. For instance, biological psychiatrists operate with the distinction between the normal and the pathological while psychoanalysts privilege the distinction between the conscious and the unconscious. What do these distinctions enable therapists to see and what do they obfuscate? What blind spots do they engender?

To give an example, while biomedical reports on clinical trials focus on the reduction of psychopathological symptoms and the restoration of mental health, they ignore that the involved psychotherapists could have worked toward additional goals such as rendering unconscious conflicts conscious, increasing the patient’s autonomy by enabling greater self-care when mental illness strikes again. Yet representatives of competing schools of psychotherapy might deem the fostering of such autonomy misguided and instead employ psychedelics to dissolve the patient’s ego because it stands in the way of her “inner healing intelligence” (Davis, 2022). Shedding light on the divergent psychological theories and ethical perspectives that inform clinical work would make the field of psychedelic therapy more transparent and enable critical reflection on values underlying alternative approaches. The psychedelic renaissance could profit from being sociologically enlightened.

There is no need to tie the psychedelic humanities to the specifics of Luhmann’s conceptual framework. There are many other frameworks available, which are less schematic and allow for empirically richer observations of how other observers observe the world. Some have found their way into the scientific literature on psychedelics, for example, when researchers present the resumption of psychedelic-assisted psychotherapy as a “paradigm shift.” (Schenberg, 2018) Philosopher of science Thomas Kuhn had introduced this notion to analyze fundamental changes in the basic concepts and research practices in a scientific discipline (Kuhn, 1962). Plain historiography or ethnography that refrains from theorizing its material the way Kuhn did also reveals differences in perspective. Take Erika Dyck’s history of psychedelic psychiatry, which contrasts the field’s initial enthusiasm in the 1950s with its demise in the 1960s (Dyck, 2008), my account of how the preclinical phase of the revival of psychedelic research differed from the first wave of psychedelic research studied by Dyck (Langlitz, 2012a), Ido Hartogsohn’s book on how the collective set and setting and thus the psychedelic experience changed over the course of the 20th century in the United States (Hartogsohn, 2020), or Claudia Schwarz-Plaschg’s sociological comparison of how the uses of psychedelics are imagined in biomedical, legal, and religious contexts (Schwarz-Plaschg, 2022). What these studies have in common is that they demonstrate that different historical, cultural, and disciplinary contexts enable different perspectives on psychedelics and that the contemporary biomedical perspective represents one possibility among several. And ethnographic research on the contemporary biomedical perspective reveals that there is even more difference within: psychedelic psychiatry is not monolithic but allows for a variety of competing perspectives and applications, which are enabled and constrained by the specific substances and the standpoints of their observers and users (Langlitz, 2012a, 2022). By showing that representations and reality are not as tightly coupled as they seem, alternative forms of representation become conceivable. For example, placebo-controlled trials might reveal one side of psychedelic drug action, culture-controlled trials reveal another (Langlitz, 2012b). Psychedelic humanities depart from the recognition that reasonable people can disagree and that their views of what knowledge counts as rational and empirical depend on their socialization and social field as well as the cultural and historical context in which they work. Their contribution to the revival of psychedelic research is to sharpen actors’ sense of contingency and possibility.

## Possibility, not normativity

Under the banner of “psychedelic studies,” literary scholar Neşe Devenot proposed to expand psychedelic research from medicine and anthropology to philosophy and the humanities. The best model for such a post-disciplinary field would be queer studies, she argued. After all, queer is whatever is at odds with the normal, the legitimate, and the dominant – and the “psychedelic identities” that people formed around drug-induced states of mind were just as deviant and oppressed as a traditionally queer lifestyle. Like queer studies, psychedelic studies of Devenot (2013) contribute to an emancipatory project that advances the moral principle of equality by examining the world from the point of view of the oppressed. Inducing such perspectival shifts that align scholarship with political causes has become a widely adopted approach in the humanities, including the psychedelic humanities. Insofar as it recognizes that knowledge depends on the position of the knower, this form of academic *littérature engagée* is one way of contributing to the Sociological Enlightenment.

Another way for the psychedelic humanities would be to drop the impulse of critique and moral betterment. There is little evidence that obtaining postgraduate degrees in the humanities makes people more moral than their fellow citizens (Fish, 2008; Schwitzgebel, 2009). Moreover, moralizing an issue makes all further communication difficult because both sides claim the moral high ground and disparage the other as immoral. Even though psychedelic research has so far escaped the politicization that haunts climate science in the Anthropocene or epidemiology in the COVID-19 pandemic, the field’s inside temperature has risen markedly in recent years. Morally charged debates over intellectual property rights and the cultural appropriation of indigenous knowledge have ravaged social relations in the so-called psychedelic community. Everything can be cast as a moral issue, nothing has to be – and often it is better to adopt a different perspective and ask if a scientific finding is true or false, or whether a new pharmaceutical formulation is new enough to be patentable. Luhmann once quipped that it was the most pressing task of ethics to warn against morality (Luhmann, 1991). Here, *ethics* designates a theoretical reflection of morality, which not only considers the appropriateness of particular moral norms but also the appropriateness of applying *any* moral norm to judge a given situation. If the psychedelic humanities aspire to normative engagement, as many kinds of humanities scholarship do, it might be more germane to do so in the form of ethical reflection on when (not) to moralize and how else to evaluate what is happening in psychedelic research and culture (Langlitz, 2020a,b).

But ethical reflection hardly exhausts the psychedelic humanities. Literary theorist Hans Ulrich Gumbrecht argues that one key task of humanists is to render phenomena more complex, for example, by opening alternative perspectives and confronting established interpretations with inconvenient facts, improbable findings, and counterintuitive insights (Gumbrecht, 2003). Tightly integrated with the (social) scientific study of extrapharmacological factors, psychedelic humanities remind laboratory researchers that the effects observed in the kind of people who participate in their more or less controlled trials are not necessarily the effects to be observed as psychedelics are used in very different settings and practices by people with very different mindsets. For example, in the face of scientific studies suggesting that psychedelics promote left-wing, anti-authoritarian, and pro-environmental attitudes, the historical and ethnographic archive presents cases of right-wing and authoritarian uses of psychedelics (Piper, 2015; Nour et al., 2017; Langlitz, 2020c; Pace and Devenot, 2021). While the denunciation of rightist currents in psychedelia reduces moral complexity by discriminating between good and bad applications, the discovery of the cultural plasticity and political pluripotency of psychedelics increases epistemic complexity by showing that psychedelic drug action can be complicated by social factors, which require both moral and psychopharmacological inquiry (Langlitz et al., 2021).

Increasing complexity is no good in itself (taking action, whether politically or clinically, requires the simplification of choices). But it does present actors with additional choices that had not been available to them: metrics to evaluate the effects of psychedelics beyond the opposition of the normal and the pathological; methodologies to study the drugs’ transformative powers beyond placebo-controlled trials; Research & Development practices that extend drug design from molecules to the extrapharmacological factors that modulate drug action. The latter might include writing philosophical essays that shape psychedelic experiences in the 21st century as profoundly as Aldous Huxley’s *The Doors of Perception* shaped psychedelic experiences in the 20th century (Huxley, 1954). The goal of the psychedelic humanities is to sharpen the sense of possibility and expand the imagination of the psychedelic renaissance.

Such a sense of possibility allows researchers to gain some distance from reality to think up other realities, which attunes them to both new opportunities and risks. As psychedelics come to be used in more diverse settings, their transformative potential will be harnessed to very different therapeutic and ethical projects (and many, maybe all therapeutic projects have an ethical subtext). A better understanding of this complexity enables researchers to imagine novel contexts of use for a class of drugs that is known to work in a highly context-dependent manner and could facilitate a burst of badly needed innovation in psychopharmacology (Langlitz, 2022). It also protects against a complacent attitude that assumes that effects observed in one historical or cultural context will necessarily be observed in other contexts. Taken at the retreat of a pharmaceutical start-up in 2022, LSD might still alter a user’s sociopolitical outlook but not in the same way as when it was taken at the Woodstock Music Festival in 1969. Recognizing the contingency of normative commitments, including one’s own, can civilize debate in any social field over where it should be heading – an insight that could benefit the psychedelic renaissance as it is becoming increasingly contentious.

## The psychedelic humanities lab

In the anglophone world, many universities have responded to the growing scientific and public interest in psychedelic drugs by creating research centers dedicated to their investigation. For the most part, these initiatives focus on clinical and preclinical research. In light of the above plea for psychedelic humanities, psychedelic research centers would be well advised to create positions for resident humanists, not cordoned off into separate research groups but closely collaborating with neuroscientists and psychiatrists. The work that philosopher Chris Letheby or cultural anthropologist David Dupuis have conducted with brain researchers and clinical psychologists uses neuroscientific data to develop a theory of how psychedelics work (Timmermann et al., 2021, 2022). Ethnographically informed and philosophically oriented research in neuropsychopharmacology laboratories offers a different model that feeds second-order observations of psychedelic research back into the research process (Langlitz, 2010, 2012a; Hendy, 2022). Yet another model would integrate bioethicists in laboratories and clinical trials: instead of evaluating the social consequences that psychedelics might have “downstream” from science on society, normatively engaged humanities scholar could get involved “upstream” in the design of novel applications (Rabinow and Bennett, 2012; Earp and Yaden, 2021; Langlitz et al., 2021). This article is not the place to provide a systematic review of projects already underway. If conducive institutional conditions are created, many more will be invented in the next years. Considering the simultaneously challenging and culturally creative social history of psychedelics, the revival of psychedelic research might find a good example in the Human Genome Project, which spent 5% of its budget on the ethical, legal, and social implications (ELSI) of genomic science. However, facing the peculiar cultural plasticity of the drugs’ psychotropic effects, psychedelic research cannot simply fall back on the ELSI program but must develop novel forms of collaborative research that aligns natural science, social research, and humanities scholarship.

At The New School, we have opened the first laboratory that studies psychedelics in their social and cultural contexts from a humanist perspective. In the wake of the “laboratory turn” in the humanities, the Psychedelic Humanities Lab brings together researchers and students with different disciplinary skillsets to collaborate on psychedelic research projects that cut across the ontological divide of nature and culture (Breithaupt, 2017; Pawlicka-Deger, 2020). Since its inception in the 19th century, the underlying metaphysics that imagined human things as separate from natural things has lost purchase in a world where *Homo sapiens* alters the climate by burning fossil fuels and his mind by ingesting psychotropics. And yet the institutionalization of this outdated order of being in separate Faculties of Art and Faculties of Science perpetuates the corresponding order of knowledge. In recent decades, we have seen many attempts at bridging the gap between the sciences and the humanities, from sociobiology to multispecies ethnography and from neurophilosophy to critical neuroscience. Considering the cultural plasticity of their neurobiological effects, psychedelics offer an opportunity for conducting experiments not just with the drugs themselves but also with the research practices through which we come to know their effects. Psychedelic humanities serve as a platform to rethink both neuropsychopharmacology and the literature concerned with human culture.

The work of the Psychedelic Humanities Lab starts from the assumption that psychedelics pose socially inflected questions of meaning and value that are independent of their therapeutic value. While the clinical efficacy and marketability of psychedelic drugs will significantly affect their (re-)introduction into mainstream science and society, the lab has no stakes in their successful medicalization or commercialization but seeks to inform the debate over both medical and nonmedical perspectives. It aims at apprehending our time in thought and understanding what difference the psychedelic renaissance introduces with respect to the first wave of psychedelic research. It analyzes how psychedelics shape ideologically very different ethical and political projects, ranging from different brands of mysticism, neoanimism, ecofeminism, radical humanism, anti-racism, and alt-right ideology. These competing normative frameworks shape the varieties of psychedelic experience today and might be amplified by the use of drugs that can increase suggestibility and induce a so-called noetic feeling of gaining direct knowledge of something grand or important about reality. Their unique psychopharmacological effects make psychedelics a double-edged sword that requires careful social scientific analysis and philosophical reflection (Timmermann et al., 2022). Such work contributes to a theoretical understanding of the cultural and historical plasticity of psychedelics. Fostering second-order observation, the Psychedelic Humanities Lab also draws attention to the contingency of any conceptual framework to keep the theoretical imagination supple and rooted in the multifariousness of human life. It understands the humanities as an inherently diverse and dissensual field (Márkus, 1987). Therefore, it does not promote any one theoretical or normative agenda in particular but the Socratic ideal of following the argument where it leads, unbridled by received wisdom or practical, social, and political implications of the conclusions reached. In the emergent field of psychedelic humanities, cultivating such a willingness to freely stay on the move between alternative points of view represents one of several possibilities.

What good is such noncommitment? It temporarily relaxes ingrained ways of thinking and doing and enables people to subsequently think and do things differently. Supposedly, this is also what psychedelics are good for (Pollan, 2018; Carhart-Harris and Friston, 2019). It may be argued that it is specifically this mind-loosening quality that makes this brand of humanities psychedelic.

## Data availability statement

The original contributions presented in the study are included in the article/supplementary material, further inquiries can be directed to the corresponding author.

## Author contributions

The author confirms being the sole contributor of this work and has approved it for publication.

## Conflict of interest

The author declares that the research was conducted in the absence of any commercial or financial relationships that could be construed as a potential conflict of interest.

## Publisher’s note

All claims expressed in this article are solely those of the authors and do not necessarily represent those of their affiliated organizations, or those of the publisher, the editors and the reviewers. Any product that may be evaluated in this article, or claim that may be made by its manufacturer, is not guaranteed or endorsed by the publisher.
